# Purification of an Antifungal Peptide from Seeds of *Brassica oleracea* var. *gongylodes* and Investigation of Its Antifungal Activity and Mechanism of Action

**DOI:** 10.3390/molecules24071337

**Published:** 2019-04-04

**Authors:** Caicheng Wang, Yao Zhang, Weiwei Zhang, Susu Yuan, Tzibun Ng, Xiujuan Ye

**Affiliations:** 1 State Key Laboratory of Ecological Pest Control for Fujian and Taiwan Crops, Fujian Agriculture and Forestry University, Fuzhou 350002, China; mustardboat@gmail.com (C.W.); zyao797@gmail.com (Y.Z.); weicheung1993@gmail.com (W.Z.); 2140215002@fafu.edu.cn (S.Y.); 2 Key Laboratory of Biopesticide and Chemical Biology, Ministry of Education, Fujian Agriculture and Forestry University, Fuzhou 350002, China; 3 Fujian Key Laboratory of Plant Virology, Institute of Plant Virology, Fujian Agriculture and Forestry University, Fuzhou 350002, China; 4 School of Biomedical Sciences, Faculty of Medicine, The Chinese University of Hong Kong, Hong Kong 999077, China; b021770@mailserv.cuhk.edu.hk

**Keywords:** *Brassica oleracea* var. *gongylodes*, antifungal peptide, purification

## Abstract

In this study, a 8.5-kDa antifungal peptide designated as BGAP was purified from the crude extract of the seeds of *Brassica oleracea* var. *gongylodes* by employing a protocol that comprised cation exchange chromatography on SP-Sepharose, cation exchange chromatography on Mono S and gel filtration chromatography on Superdex peptide. BGAP showed the highest amino acid sequence similarity to defensin peptides by mass spectrometric analysis. BGAP showed a broad spectrum of antifungal activity with a half maximal inhibitory concentration at 17.33 μg/mL, 12.37 μg/mL, 16.81 μg/mL, and 5.60 μg/mL toward *Colletotrichum higginsianum*, *Exserohilum turcicum*, *Magnaporthe oryzae* and *Mycosphaerella arachidicola*, respectively. The antifungal activity of BGAP remained stable (i) after heat treatment at 40–100 °C for 15 min; (ii) after exposure to solutions of pH 1–3 and 11–13 for 15 min; (iii) after incubation with solutions containing K^+^, Ca^2+^, Mg^2+^, Mn^2+^ or Fe^3+^ ions at the concentrations of 20–150 mmol/L for 2 h; and (iv) following treatment with 10% methyl alcohol, 10% ethanol, 10% isopropanol or 10% chloroform for 2 h. Fluorescence staining experiments showed that BGAP brought about an increase in cell membrane permeability, a rise in reactive oxygen species production, a decrease in mitochondrial membrane potential, and an accumulation of chitin at the hyphal tips of *Mycosphaerella arachidicola*.

## 1. Introduction

Plants are often attacked by fungi during growth. Infection by pathogenic fungi produces a deleterious effect on plant growth and may even cause death, ensuing in a plummeting of grain yield [1,2]. The release of certain mycotoxins during the infection process poses a tremendous threat to the safety of agricultural products [3,4]. Although various plant protection strategies such as comprehensive management and ecological management have been proposed, in practice, chemical control remains as the main preventive and control measure for plant diseases [5,6]. Although chemical agents demonstrate certain control effects on a variety of phytopathogenic fungi, the current practice of the use of chemicals is marred by drawbacks. It not only brings about serious environmental pollution and gives rise to the presence of the residues of these chemicals in agricultural products [7], it also aggravates the issue of fungicide resistance [5,8]. Thus, it is of utmost importance to screen for new antifungal substances characterized by safety, high efficiency, environmental friendliness, and lower fungicide resistance. Among the candidates, antifungal peptides have considerable application potential and attracted attention [9,10,11,12].

Antifungal activity has been detected in a diversity of living organisms including microbes, animals and plants, and antifungal peptides have been isolated from them [13,14,15]. For the sake of safety and availability, in our search for antifungal peptides, we have looked more at plants that have long been used as foods. Antifungal peptides, which are important components of plant defense systems, are distributed in the fruits, flowers, leaves, tubers, radicles, and seeds of plants [13,16,17,18,19,20,21], among which peptides derived from seeds are more common. At the early stage of this research, we performed assays of antifungal activity on crude protein extracts from various plant seeds and discovered that the crude protein extract of Kohlrabi (*Brassica oleracea* var. *gongylodes*) seeds exhibited good antifungal activity against various phytopathogenic fungi. Consequently, in this study, we purified and characterized the antifungal peptide from Kohlrabi seeds extract, studied its antifungal activity and stability, and investigated its effects on pathogenic fungi, with the intent to provide a basis for further unraveling the mechanism of action of the antifungal peptide and broadening the scope of its application.

## 2. Results

**Figure 1:**
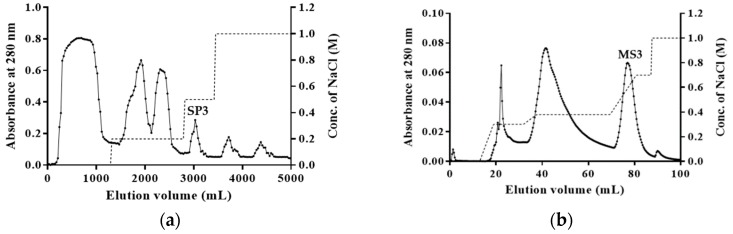

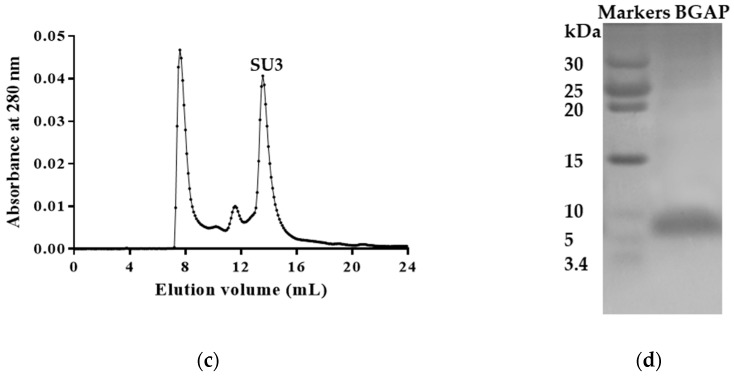
Purification and molecular weight determination of BGAP: (**a**) cation exchange chromatography of crude protein extract on SP-Sepharose column: (−): absorbance at 280 nm, (…): concentration of NaCl; (**b**) cation exchange chromatography of active fraction SP3 from SP-Sepharose column on Mono S column; (**c**) gel filtration chromatography of active fraction MS3 from Mono S column on Superdex peptide column; (**d**) Tricine-SDS-PAGE of active fraction SU3 from Superdex peptide column.

Fraction SP3 with antifungal activity was obtained by cation exchange chromatography of the crude protein extract of Kohlrabi seed extract on SP-Sepharose (Figure 1a). The active fraction MS3 was obtained from fraction SP3 by cation exchange chromatography on Mono S (Figure 1b). The active fraction SU3 was obtained from fraction MS3 by gel filtration chromatography on Superdex peptide (Figure 1c). Fractions not mentioned above did not have antifungal activity. Fraction SU3 contained a highly-purified antifungal peptide, which was named BGAP which showed a molecular weight of about 8.5 kDa in SDS-PAGE (Figure 1d) and was found to have reliable similarity with defensins and thionins by MALDI-TOF/TOF MS analysis, and had the highest similarity with defensin-like protein 1 from *Brassica oleracea* var. *oleracea* (Table 1). The defensin peptides listed in the table are peptides predicted by genomic sequences; the details can be obtained from the protein database of the NCIB database according to the accession No. provided in Table 1.

**Figure 2:**
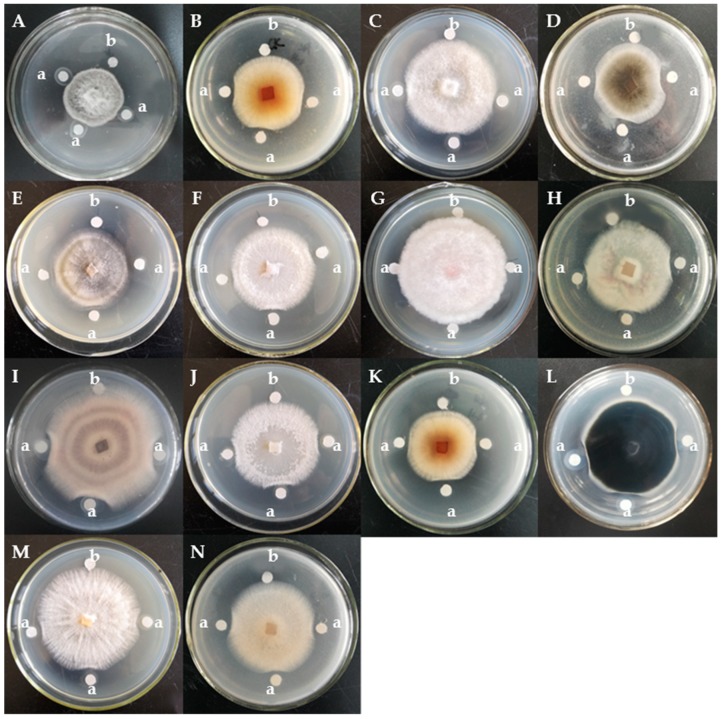
Growth inhibitory effects of BGAP represented by fraction SU3 toward 14 phytopathogenic fungi: (**A**) *Alternaria longipes*; (**B**) *Botrytis cinerea*; (**C**) *Colletotrichum gloeosporioides*; (**D**) *Colletotrichum higginsianum*; (**E**) *Colletotrichum micotianae*; (**F**) *Exserohilum turcicum*; (**G**) *Fusarium graminearum*; (**H**) *Fusarium oxysporum*; (**I**) *Fusarium solani* f. sp. *glycines*; (**J**) *Helminthosporium maydis*; (**K**) *Magnaporthe oryzae*; (**L**) *Mycosphaerella arachidicola*; (**M**) *Pestalotiopsis microspora*; and (**N**) *Valsa mali*; a: 20 μL of BGAP solution (1 mg/mL); b: 20 μL of PBS buffer.

**Figure 3:**
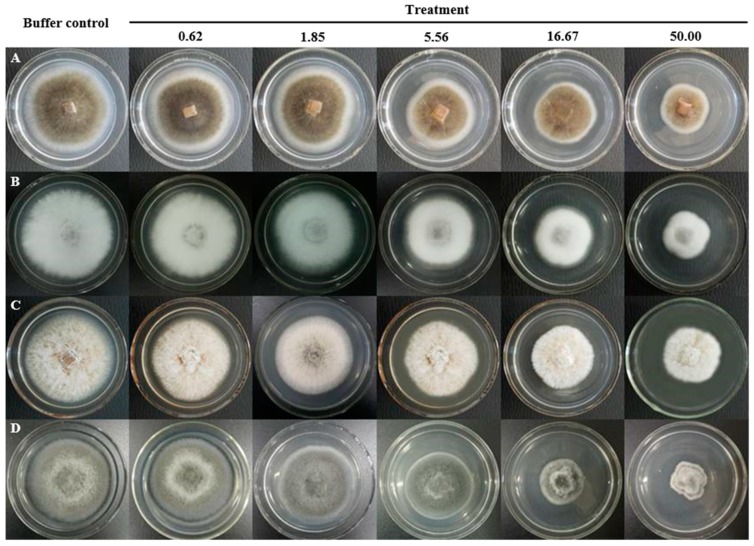
IC_50_ determination of BGAP toward four phytopathogenic fungi: (**A**) *Colletotrichum higginsianum*; (**B**) *Exserohilum turcicum*; (**C**) *Magnaporthe oryzae*; and (**D**) *Mycosphaerella arachidicola*; numerical value represents the concentration (μg/mL) of BGAP in the medium.

**Figure 4:**
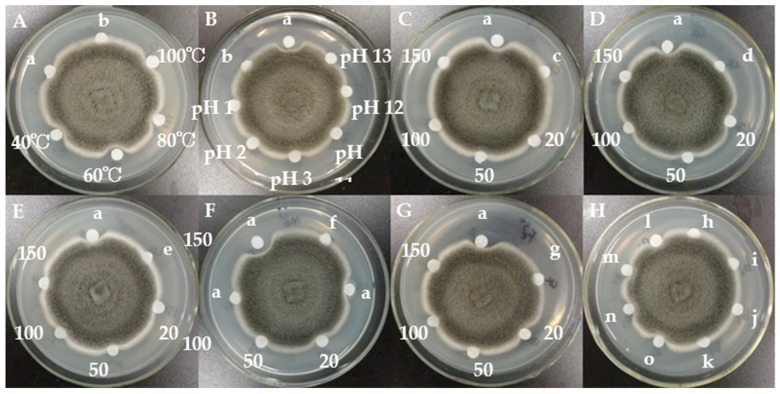
Effects of heat, acid-base, metal ions, and organic solvents on antifungal activity of BGAP: (**A**) heat treatment; (**B**) acid-base treatment; (**C**) treatment with K^+^ ions; (**D**) treatment with Ca^2+^ ions; (**E**) treatment with Mg^2+^ ions; (**F**) treatment with Mn^2+^ ions; (**G**) treatment with Fe^3+^ ions; (**H**) organic solvent treatment; a: 20 μL of untreated BGAP solution; b: 20 μL of PBS buffer; c: 20 μL of K^+^ solution (150 mmol/L); d: 20 μL of Ca^2+^ solution (150 mmol/L); e: 20 μL of Mg^2+^ solution (150 mmol/L); f: 20 μL of Mn^2+^ solution (150 mmol/L); g: 20 μL of Fe^3+^ solution (150 mmol/L); h: 20 μL of 10% methanol; i: 20 μL of 10% ethanol; j: 20 μL of 10% isopropanol; k: 20 μL of 10% chloroform; 40–100 °C represents 20 μL of BGAP solution treated at the corresponding temperature; pH 1–13 represents 20 μL of BGAP solution treated with the corresponding acid-base; 20–150 represents 20 μL of BGAP solution treated with each metal ion at a concentration of 20–150 mmol/L; l, m, n, o represent BGAP solution treated with 10% methanol, 10% ethanol, 10% isopropanol and 10% chloroform respectively; and the final concentration of BGAP in all treatment groups was 1 mg/mL.

The assay of antifungal activity disclosed that BGAP manifested potent growth inhibitory activity against the following 12 phytopathogenic fungi encompassing *Alternaria longipes*, *Botrytis cinerea*, *Colletotrichum gloeosporioides*, *C. higginsianum*, *Exserohilum turcicum*, *Fusarium oxysporum*, *F. solani* f. sp. *glycines*, *Helminthosporium maydis*, *Magnaporthe oryzae*, *Mycosphaerella arachidicola*, *Pestalotiopsis microspora,* and *Valsa mali*, but expressed only weak activity against *F. graminearum* and *C. micotianae* (Figure 2). BGAP was devoid of growth retarding effect on the eight bacteria tested including *Bacillus subtilis*, *Erwinia carotovora*, *Escherichia coli*, *Pseudomonas aeruginosa*, *P. pseudoalcaligenes*, *Ralstonia solanacearum*, *Salmonella* sp., and *Staphylococcus aureus*. The determination of half maximal inhibitory concentration (IC_50_) was performed (Figure 3). The IC_50_ values of BGAP toward *Colletotrichum higginsianum*, *Exserohilum turcicum*, *Magnaporthe oryzae,* and *Mycosphaerella arachidicola* were 17.33 μg/mL, 12.37 μg/mL, 16.81 μg/mL, and 5.60 μg/mL, respectively. The pronounced stability of the antifungal activity of BGAP was observed after heat treatment at 40–100 °C for 15 min (Figure 4A); after treatment with acid-base solutions of pH 1–3 and 11–13 for 15 min (Figure 4B); following exposure to solutions containing K^+^, Ca^2+^, Mg^2+^, Mn^2+^ or Fe^3+^ ions at 20–150 mmol/L for 2 h (Figure 4C–G), and after treatment with methanol, ethanol, isopropanol, and chloroform at a concentration of 10% (Figure 4H).

**Figure 5:**
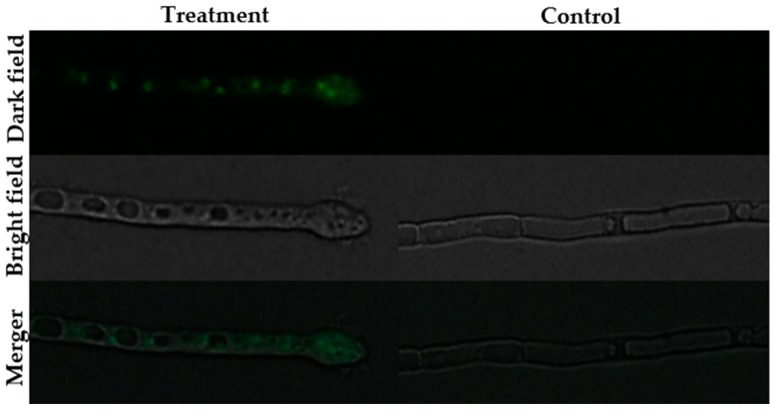
Permeabilization of *Mycosphaerella arachidicola* hyphal membrane caused by BGAP.

**Figure 6:**
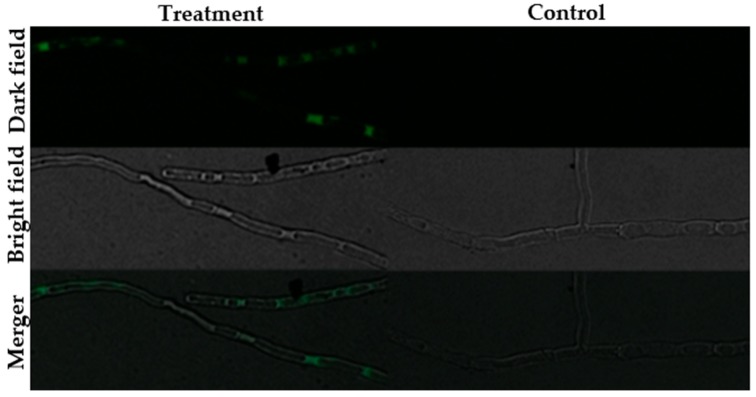
Increase in ROS production of *Mycosphaerella arachidicola* caused by BGAP.

**Figure 7:**
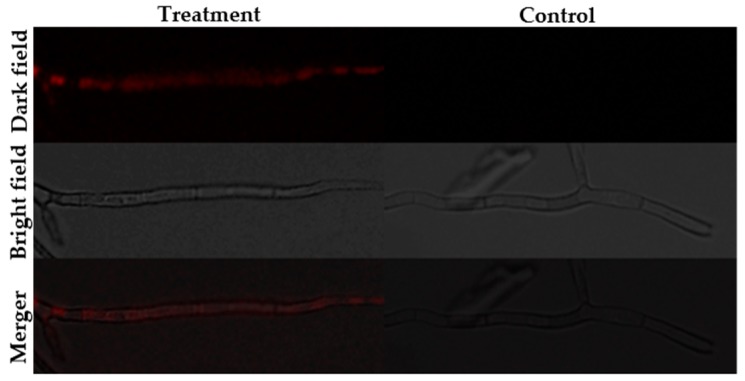
Decrease in mitochondrial membrane potential of *Mycosphaerella arachidicola* caused by BGAP.

**Figure 8:**
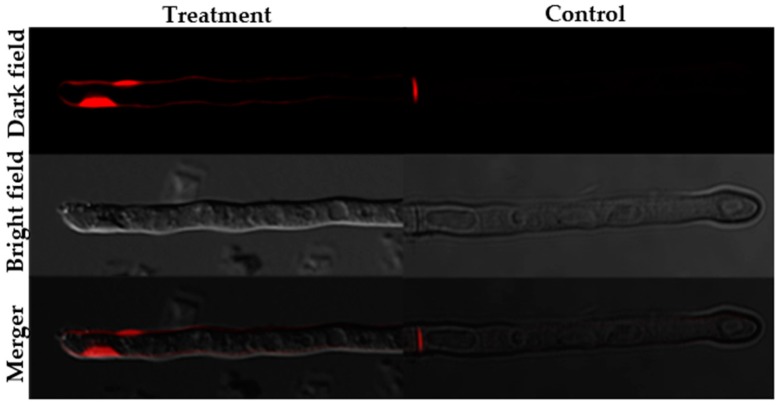
Accumulation of chitin at the hyphal tips of *Mycosphaerella arachidicola* caused by BGAP.

The fluorescence staining experiments disclosed that following staining with SYTOX green, H_2_DCFDA and Rhodamine 123, fluorescence was observed in the hyphae of the BGAP-treated group, but not in the hyphae of the control group (Figure 5, Figure 6 and Figure 7). After staining with Congo red, the fluorescence intensity at the hyphal tips of the BGAP-treated group was significantly stronger than that of the control group (Figure 8), indicating that BGAP engendered an increase in cell membrane permeability, a rise in reactive oxygen species (ROS) production, a decline in mitochondrial membrane potential, and accumulation of chitin at the hyphal tips of *Mycosphaerella arachidicola*.

## 3. Discussion

The results of mass spectrometric analysis disclosed that BGAP was structurally similar to both defensins and thionins; therefore, we have listed the defensin peptides and thionin peptides recently isolated from plants in Table 2. In terms of antimicrobial activity, BGAP exhibits some similarities and differences compared with them. Among defensin-like peptides, Tf-AFP inhibited growth in *Fusarium oxysporum* [19]. Large pinto bean defensin exerted an inhibitory action on *Fusarium oxysporum* and *Mycosphaerella arachidicola* [21]. Legumi secchi peptide displayed inhibitory activity against *Fusarium oxysporum*, *Helminthosporium maydis* and *Mycosphaerella arachidicola* [22]. Limyin demonstrated inhibitory activity against *Botrytis cinerea* [23]. BGAP also evinced inhibitory activity against the above pathogenic fungi. On the contrary, limyin did not exhibit significant suppressive activity on *Staphylococcus aureus* and *Salmonella* sp. Similarly, BGAP did not have antibacterial activity against the eight bacterial species tested including Gram-positive and Gram-negative bacteria. Both large pinto bean defensin and the legumi secchi peptide were devoid of suppressive activity on *Valsa mali*. In contrast, BGAP inhibited *Valsa mali*, indicating that BGAP has a wider spectrum of antifungal action. Thionin-like peptides inhibited and killed fungi as well as Gram-positive and Gram-negative bacteria [24,25,26,27]. The antimicrobial activity of thionins could be inhibited by metal ions [28,29]. However, BGAP was devoid of antibacterial activity and also exhibited certain antifungal activity after treatment with solutions containing K^+^, Ca^2+^, Mg^2+^, Mn^2+^ or Fe^3+^ ions. From this perspective, BGAP resembles defensins more than thionins. Table 3 lists the antifungal proteins and peptides previously isolated from *Brassica* spp. The molecular weights of juncin, brassiparin and kale peptides were 18.9 kDa, 5716 Da, and 5907 Da [30,31,32], respectively, while the molecular weight of BGAP was 8.5 kDa, which was closer to those of campesin (9.4 kDa) and nsLTP (9414 Da) [33,34]. However, both campesin and nsLTP were similar in sequence to lipid transfer protein, while BGAP was structurally similar to defensin, indicating that BGAP is a peptide different from the abovementioned peptides. Nevertheless, BGAP exhibited a broad spectrum of antifungal activity and pronounced stability, which is similar to all of the antifungal proteins and peptides from *Brassica* spp. In addition, compared to previous reports, we have conducted more research on BGAP in terms of the mode of antifungal action.

BGAP may undermine the growth of fungi by enhancing cell membrane permeability, boosting the production of ROS and disrupting mitochondrial membrane potential. The action of permeabilizing the fungal cell membrane is analogous to the legium secchi peptide, NsW2, and *Capsicum annuum* peptide listed in Table 2. Membrane permeabilization may involve fusion of antifungal peptide with the cell wall and cell membrane [35,36,37]. Several models related to membrane permeabilization have been proposed, such as the barrel-stave pore model, carpet model and thoroidal pore model [38]. Excessive production of ROS and decline of mitochondrial membrane potential may further trigger apoptosis of mycelial cells, since ROS plays a central role in the regulation of apoptosis [39], and mitochondrial membrane potential acts as a marker of early apoptosis [40]. Cellular ROS accumulation can ensue in the opening of mitochondrial permeability transition pores, leading to a fall in mitochondrial membrane potential and release of cytochrome c [39,40,41]. Cytochrome c interacts with apoptotic protease-activating factor, which in turn initiates a cascade of caspase activation to bring about apoptosis [42]. BGAP can also induce chitin deposition at the mycelial tip, which is reminiscent of the action of cabbage protein, Fava bean protein VFTI-E1 and *Peltophorum ptercoarpum* antifungal amidase [43,44,45]. However, the accumulation of chitin at the tip of the mycelium may be a factor that hinders mycelial growth and may also be a countermeasure adopted by pathogenic fungi against antifungal peptides [46]. At present, it is not clear which of the above phenomena mainly accounts for the observed inhibition of fungal growth. How these phenomena are triggered and whether there are more correlations between the phenomena remain to be studied.

To recapitulate, we report herein the isolation and characterization of a potent and stable antifungal peptide with exploitable potential from the seeds of *Brassica oleracea* var. *gongylodes*.

## 4. Materials and Methods

### 4.1. Materials

Kohlrabi seeds collected in Beijing, China were purchased from a seed vendor. The SP-Sepharose, Mono S 5/50 GL and Superdex peptide 10/300 GL columns used for chromatography were purchased from GE Healthcare (Piscataway, NJ, USA). The 14 phytopathogenic fungi including *Alternaria longipes*, *Botrytis cinerea*, *Colletotrichum gloeosporioides*, *C. higginsianum*, *C. micotianae*, *Exserohilum turcicum*, *Fusarium graminearum*, *F. oxysporum*, *F. solani* f. sp. *glycines*, *Helminthosporium maydis*, *Magnaporthe oryzae*, *Mycosphaerella arachidicola*, *Pestalotiopsis microspora,* and *Valsa mali* used in the following experiments were provided by Key Laboratory of Biopesticide and Chemical Biology, Fujian Agriculture and Forestry University. Eight bacterial species comprising *Bacillus subtilis*, *Erwinia carotovora*, *Escherichia coli*, *Pseudomonas aeruginosa*, *P. pseudoalcaligenes*, *Ralstonia solanacearum*, *Salmonella* sp., and *Staphylococcus aureus* utilized in the experiments described below were obtained from the same aforementioned source.

### 4.2. Purification of the Antifungal Peptide BGAP

Seeds (150 g) were immersed overnight in 1 L of 10 mM NH_4_OAc buffer (pH 4.6), homogenized and then left at 4 °C for 12 h. The homogenate was centrifuged (10000× *g*, 4 °C, 30 min). The resulting supernatant was filtered through four layers of gauze, and the filtrate was collected to obtain a crude extract. The crude extract was chromatographed on a column (5 cm × 16 cm) of SP-Sepharose which had previously been equilibrated with 10 mM NH_4_OAc buffer (pH 4.6). After the unadsorbed proteins had been eluted from the column, the adsorbed proteins were eluted successively with 10 mM NH_4_OAc buffer (pH 4.6) containing 0.2 mol/L, 0.5 mol/L, and 1 mol/L NaCl. The active fractions with antifungal activity were dialyzed against double-distilled water in a dialysis bag with a molecular weight cut-off of 3500 Da and then freeze-dried into powder form. The protein powders were redissolved in 10 mM NH_4_OAc buffer (pH 4.6), followed by ion exchange chromatography using a Mono S 5/50 GL column attached to the AKTA Purifier system (GE Healthcare). The column had been pre-equilibrated with 10 mM NH_4_OAc buffer (pH 4.6) and the adsorbed proteins were eluted with NH_4_OAc buffer containing a linear gradient of 0-1 mol/L NaCl. The active fractions were collected, dialyzed, lyophilized, and redissolved in NH_4_OAc buffer and then subjected to gel filtration chromatography using a Superdex peptide 10/300 GL column attached to the AKTA Purifier system (GE Healthcare, Piscataway, NJ, USA). The absorption peaks SP3, MS3, and SU3 obtained from the various stages of chromatography contained an antifungal peptide with gradually increasing purity.

### 4.3. Molecular Weight Determination by Tricine-SDS-PAGE, Mass Spectrometry and Determination of Concentration of Antifungal Peptide

The molecular weight of the antifungal peptide was determined by tricine-sodium dodecyl sulfate-polyacrylamide gel electrophoresis (tricine-SDS-PAGE) with a slight modification of the method provided by Schagger [47]. Briefly, the antifungal peptide sample was mixed with loading buffer containing 24% glycerol, 3% β-mercaptoethanol, 10% SDS, 0.02% Coomassie brilliant blue G-250 and 0.1 mol/L Tris buffer (pH 6.8) in a ratio of 4:1 (vol:vol), followed by boiling in water for 5 min. The supernatant was removed for electrophoresis. In the electrophoresis, 4% stacking gel, 10% spacer gel and 16.5% separating gel were used, and gel staining was performed by employing the Coomassie brilliant blue method. After the gel had been destained, the antifungal peptide band in the gel was excised and sent to Shanghai Applied Protein Technology Co., Ltd (Shanghai, China) for matrix-assisted laser desorption/ionization with time-of-flight/time-of-flight mass spectrometry (MALDI-TOF/TOF MS) by using a 5800 MALDI-TOF/TOF mass spectrometric instrument (AB SCIEX, Framingham, MA, USA). Briefly, the sample in the gel was enzymatically hydrolyzed in a sequencing grade trypsin solution at 37 °C for 20 h. After the enzymatic hydrolysate was removed, 0.1% TFA in 60% acetonitrile was added to the gel and sonicated for 15 min. Subsequently, the enzymatic hydrolysate was combined and desalted by using a ZipTip tip. The sample solution (1 μL) was added to the sample target and allowed to dry naturally, and then 0.6 μL of supersaturated CHCA matrix solution (dissolved in 0.1% TFA in 50% acetonitrile) was added, dried naturally, and analyzed by employing a MALDI-TOF-TOF analyzer. The information regarding the resulting peptide fragments was subjected to a database search using Mascot 2.2 software against NCBInr databases. The peptide concentration used in all experiments was measured with the BCA method using a kit.

### 4.4. Assay of Antifungal Activity

Pathogenic fungi were inoculated on a PDA medium plate (90 mm × 15 mm culture dish) and cultured at 28 °C. When each fungal mycelium had grown to a diameter of 4–5 cm, four sterile filter paper disks, each with a diameter of 6 mm, were placed 5 mm from the periphery of the mycelial colony. An antifungal peptide solution at a concentration of 1 mg/mL was prepared in 20 mmol/L phosphate buffered saline (PBS) buffer (pH 7.0). Then 20 μL of the antifungal peptide solution were added to each of the three paper disks, and another 20 μL of PBS buffer was added to the fourth paper disk as a blank control. After the treatment, the plates were continuously cultured at 28 °C, and the growth status of each fungal mycelium was observed within 2–3 days [21].

### 4.5. Assay of Antibacterial Activity

Activated bacteria were inoculated on LB slant medium and cultured at 37 °C for 20 h. The bacterial suspension was prepared by washing the lawn with a 0.9% (*w*/*v*) NaCl solution, and the OD_600_ value of the bacterial suspension was adjusted to 1.0. LB medium (15 mL) was poured into the petri dish, and sterile Oxford cups (8 mm × 10 mm) were placed on the plate after solidification. A 1 mL aliquot of the bacterial suspension was added to 14 mL of LB medium (0.7% agar), cooled to about 50 °C, mixed well, poured into a plate, and after it had solidified, the Oxford cups were taken out. Fifty microliters of antifungal peptide solution (1 mg/mL) were added to each of the three wells in each plate, and 50 microliters of PBS buffer was added to the fourth well as a control. The treated plate was placed at 4 °C for 6 h and then transferred to an incubator at 37 °C. After 16 h of culture, the inhibition zone on the plate was observed.

### 4.6. Assay of Stability of Antifungal Activity to Temperature Changes, pH Changes and in Presence of Metal Chlorides

An antifungal peptide solution at a concentration of 1 mg/mL was prepared in 20 mmol/L PBS buffer, and 100 μL of antifungal peptide solution was placed in a water bath at 40–100 °C for 15 min. Then the assay was performed to determine the stability of antifungal activity to heat treatment. An antifungal peptide solution at a concentration of 1 mg/mL was prepared in distilled water and was freeze-dried to dry powder. The peptide powder was redissolved in PBS buffer at pH 1–3 and pH 11–13, and placed at 25 °C for 15 min. Then the assay of antifungal activity was performed to determine the stability of the antifungal peptide to acid-base treatment. An antifungal peptide solution at a concentration of 2 mg/mL was prepared in 40 mmol/L PBS buffer, and 50 μL of the solution was added to 50 μL of a solution containing 150 mmol/L KCl, 150 mmol/L CaCl_2_, 150 mmol/L MgCl_2_, 150 mmol/L MnCl_2_ or 150 mmol/L FeCl_3_. Then the assay of antifungal activity was performed to determine the stability of antifungal activity to treatment with metal ions after the mixture had been incubated at 25 °C for 2 h. An antifungal peptide solution (concentration 2 mg/mL) was prepared in 40 mmol/L PBS buffer, and 50 μL of antifungal peptide solution was added to 50 μL of solutions containing methanol, ethanol, isopropanol and chloroform at a concentration of 10% (*v*/*v*). Then the assay of antifungal activity was performed to determine the stability of antifungal activity to treatment with various organic solvents after incubation of the mixture at 25 °C for 2 h. In the above experiments, *Mycosphaerella arachidicola* was used as the test fungus.

### 4.7. Assay of Antifungal Activity Based on the Half-maximal Inhibitory Concentration

A solution of the antifungal peptide at a concentration of 250 μg/mL was prepared in PBS buffer and serially diluted three times. One milliliter of the antifungal peptide solution filtered through a 0.22 μm sterile filter was added to 4 mL of PDA medium cooled to about 50 °C, and mixed and poured into a petri dish (55 mm × 15 mm). A 5-mm diameter of the fungus was cut at the edge of the activated fungal colony using a sterilizing punch and inoculated on the above plate. The inoculated plate was cultured at 28 °C, and the diameter of the mycelial colony of the pathogenic fungus was measured every 12 h [45]. In the control group, the antifungal peptide solution was replaced by the same volume of PBS buffer, and there were three replicates in each of the treatment groups and the control group. The fungi tested included *Colletotrichum higginsianum*, *Exserohilum turcicum*, *Magnaporthe oryzae* and *Mycosphaerella arachidicola*. The growth inhibition rate was calculated using the following formula: (inhibition of fungal growth = (1 − mycelial colony area of treatment group/mycelial colony area of control group) × 100%). The half maximal inhibitory concentration of the antifungal peptide against each pathogenic fungus was calculated by utilizing the GraphPad Prism 6 software (GraphPad Software, Inc., San Diego, CA, USA).

### 4.8. Assays of Cell Membrane Permeability Changes, Reactive Oxygen Species Production, Mitochondrial Membrane Potential Changes and Chitin Accumulation in the Fungus Mycosphaerella arachidicola

The fungal hyphae cultured in medium containing the antifungal peptide were stained with the fluorescent dyes SYTOX green, H_2_DCFDA, Rhodamine 123, and Congo red, respectively, to perform assays of cell membrane permeability change, reactive oxygen species accumulation, mitochondrial membrane potential change, and chitin accumulation, respectively [22,45,48,49,50]. *Mycosphaerella arachidicola* was used as the test fungus. The test fungus was inoculated into PD liquid medium, and the antifungal peptide was added to a final concentration of 1 mg/mL after culture with shaking (150 rmp/min) at a constant temperature (28 °C) for 2 days. The same volume of PBS buffer was added in the control group. The hyphae used for SYTOX green, H_2_DCFDA and Rhodamine 123 staining were collected after 12 h of culture, and the hyphae used for Congo red staining was collected after 24 h of cultivation. The hyphae were resuspended in 500 μL of PBS buffer, then mixed with 1 μL of fluorescent dye and stained for 30 min in the dark. After excess stain had been removed by washing with PBS buffer, the hyphae were picked to prepare smears, and the staining results were observed under a fluorescence microscope. Among the above fluorescent dyes, SYTOX green is a high-affinity nucleic acid dye that can enter cells when the plasma membrane is damaged, and the concentration used in the experiment was 5 mmol/mL. H_2_DCFDA is a cell osmotic indicator of ROS that fluoresces after deacetylation by intracellular esters and oxidation by ROS, and the concentration used in the experiment was 2 mg/mL. Rhodamine 123 is a cationic fluorescent dye that can penetrate cell membranes. It can rely on the mitochondrial transmembrane potential to enter the mitochondrial matrix in normal cells. Then the fluorescence intensity will become attenuated or disappear. The concentration of Rhodamine 123 used in the experiment was 2 mg/mL. Congo red can be tightly bound to chitin, and the concentration used in the experiment was 1.5 mg/mL.

## Figures and Tables

**Table 1:** MALDI-TOF/TOF MS results of various peptides derived from BGAP.

Protein/Peptide	Accession No.	Species of Origin	Mol wt	Sequence	Protein Score	Protein Score C.I. %
defensin-like protein 1	XP_013619391.1	*Brassica oleracea* var. *oleracea*	8694.2 Da	MAKFASIIALLFAALVLFAALEAPTMVEAQKLCERPSGTWSGVCGNNNACKNQCINLEKARHGSCNYVFPAHKCICYFPC	112	100
thionin	BAM15659.1	*Brassica oleracea* var. *viridis*	7194.5 Da	VALLFSALVIFAAFEAPTMVEAQKLCERPSGTWSGVCGNNNACKNQCIRLEKARHGSCNYVFPAHK	89	99.99
defensin-like protein 1	XP_013590720.1	*Brassica oleracea* var. *oleracea*	8784.3 Da	MAKFASIIVLLFAALVLFAGFEAPTMVEAQKLCERPSGTWSGVCGNNNACKNQCIRLEKARHGSCNYVFPAHKCICYFPC	88	99.99
thionin	BAM15658.1	*Brassica oleracea* var. *viridis*	6328.1 Da	IVLLFAEAPTMVEAQKLCERPSGTWSGVCGNNNGCKNQGIRLEKARHGSCNYVFPAHK	83	99.97
defensin-like protein 3	XP_013590858.1	*Brassica oleracea* var. *oleracea*	8619.1 Da	MAKAATITTFLFAALVLFAAFEAPTMVDAKLCERPSGTWSGVCGNNNECKKQCIRLEGARHGSCNYVFPAHKCICYFPC	82	99.97
defensin-like protein 4	XP_013635185.1	*Brassica oleracea* var. *oleracea*	10573.2 Da	MDKATKSVSSLAAFFILFLVIFEMPEIEAQDSECLKEYGGDVGFGFCAPRIYPTFCVKRCRADKGALGGKCIWGQGSNVKCLCNFCRPEPGQILSGI	77	99.88
defensin-like protein 195	XP_013634959.1	*Brassica oleracea* var. *oleracea*	9897.7 Da	MAIKPLSIFVVFFIFFLVISDMPETEAQDSKCLREYGGDVGFGFCAPRIFPTICYTRCRENKGAKGGRCRWGQGTNVTCLCDYCNDQP	66	98.36

**Table 2:** Comparison of antimicrobial activity of BGAP with defensin-like peptides and thionin-like peptides isolated from other plants.

Type	Peptide	Species of Origin	Antimicrobial Activity Toward Fungi or Bacteria	Ref.
	BGAP	*Brassica oleracea* var. *gongylodes*	**Fungi:***Colletotrichum higginsianum* (17.33 μg/mL*^a^*), *Exserohilum turcicum* (12.37 μg/mL*^a^*), *Magnaporthe oryzae* (16.81 μg/mL*^a^*), *Mycosphaerella arachidicola* (5.60 μg/mL*^a^*), *etc.*	This study
Defensin-like peptide	Tf-AFP	*Trigonella foenum-graecum*	**Fungi:***Fusarium oxysporum*, *Fusarium solani*, *Rhizoctonia solani*	[19]
	Large pinto bean defensin	*Phaseolus vulgaris*	**Fungi:***Bipolaris maydis*, *Fusarium oxysporum*, *Mycosphaerella arachidicola*, *Setosphaeria turcica*, *Valsa mali* (no effect)	[21]
	Legumi secchi peptide	*Phaseolus vulgaris*	**Fungi:***Fusarium oxysporum* (9.2 μM*^a^*), *Helminthosporium maydis* (9.5 μM*^a^*), *Mycosphaerella arachidicola* (1 μM*^a^*), *Rhizoctonia solani* (3.5 μM*^a^*), *Valsa mali* (no effect)	[22]
	Limyin	*Phaseolus limensis*	**Fungi:***Alternaria alternata*, *Botrytis cinerea*, *Fusarium solani* (8.6 μM*^a^*), *Mycosphaerella arachidicola* (no effect), *Pythium aphanidermatum* (no effect) **Bacteria:** *Staphylococcus aureus* (no effect), *Salmonella* sp. (no effect)	[23]
Thionin-like peptide	NsW2	*Nigella sativa*	**Fungi:***Candida albicans* (1.63 μM*^a^*) **Bacteria:** *Bacillus subtilis* (1.63 μM*^a^*), *Escherichia coli* (>26.0 μM*^a^*), *Staphylococcus aureus* (3.25 μM*^a^*)	[24]
	*Capsicum annuum* peptide	*Capsicum annuum*	**Fungi:***Candida albicans* (96%*^b^*, 100 μg/mL*^c^*), *Candida tropicalis* (100%*^b^*, 100 μg/mL*^c^*), *Saccharomyces cerevisiae* (98%*^b^*, 100 μg/mL*^c^*) **Bacteria:** *Escherichia coli* (18%*^b^*, 100 μg/mL*^c^*), *Pseudomonas aeruginosa* (10%*^b^*, 100 μg/mL*^c^*)	[25]
	Cp-thionin II	*Vigna unguiculata*	**Bacteria:***Escherichia coli* (64 μg/mL*^d^*), *Pseudomonas syringae* (42 μg/mL*^d^*), *Staphylococcus aureus* (128 μg/mL*^d^*), *Erwinia* sp. (no effect), *Ralstonia solanacearum* (no effect); *Rhataybacter* sp. (no effect)	[26]
	Tu-AMP 1	*Tulipa gesneriana*	**Fungi:***Fusarium oxysporum* (2 μg/mL*^a^*), *Geotrichum candidum* (2 μg/mL*^a^*); *Agrobacterium radiobacter* (15 μg/mL*^a^*) **Bacteria:** *Agrobacterium rhizogenes* (20 μg/mL*^a^*), *Curtobacterium flaccumfaciens* (13 μg/mL*^a^*), *Clavibacter michiganensis* (14 μg/mL*^a^*), *Erwinia carotovora* (11 μg/mL*^a^*)	[27]

*^a^*: half maximal inhibitory concentration; *^b^*: growth inhibition rate; *^c^*: tested peptide concentration; *^d^*: minimum inhibitory concentration.

**Table 3:** Comparison of characteristics of BGAP with antifungal proteins and peptides isolated from *Brassica* spp.

Protein/Peptide	Species of Origin	Mol (wt)	Antifungal Activity	Stability	Reported Effect	Ref.
BGAP	*Brassica oleracea* var*. gongylodes*	8.5 kDa	*Colletotrichum higginsianum* (16.94 μg/mL), *Exserohilum turcicum* (12.30 μg/mL), *Magnaporthe oryzae* (16.45 μg/mL), *Mycosphaerella arachidicola* (5.70 μg/mL), etc.	40–100 °C; pH 1–3, pH 11–13; methanol, ethanol, isopropanol, chloroform (10%); Ca^2+^, Fe^3+^, K^+^, Mg^2+^ (20–150 mM)	Accumulation of chitin at the tip of mycelium, membrane permeabilization, change of mitochondrial membrane potential, ROS generation	This study
Juncin	*Brassica juncea* var*. integrifolia*	18.9 kDa	*Fusarium oxysporum* (13.5 μM), *Helminthosporium maydis* (27 μM), *Mycosphaerella arachidicola* (10 μM), *Candida albicans*	Not tested	No membrane permeabilization (toward *Candida albicans*) and chitin deposition (toward *Mycosphaerella arachidicola*)	[30]
Brassiparin	5716 kDa	*Brassica parachinensis*	*Fusarium oxysporum* (3.9 μM), *Helminthosporium maydis* (4.7 μM), *Mycosphaerella arachidicola* (2.6 μM), *Valsa mali* (0.22 μM)	40–100 °C; pH 1–3, pH 11–13	Not tested	[31]
Kale peptide	*Brassica alboglabra*	5907 Da	*Fusarium oxysporum* (4.3 μM), *Helminthosporium maydis* (2.1 μM), *Mycosphaerella arachidicola* (2.4 μM), *Valsa mali* (0.15 μM)	20–80 °C; pH 2–3, pH 10–11	Not tested	[32]
Campesin	*Brassica campestris*	9.4 kDa	*Fusarium oxysporum* (5.1 μM), *Mycosphaerella arachidicola* (4.4 μM)	0–100 °C; pH 0–14	Not tested	[33]
nsLTP	*Brassica campestris*	9414 Da	*Fusarium oxysporum* (8.3 μM), *Helminthosporium staivum*, *Mycosphaerella arachidicola* (4.5 μM), *Sclerotinia sclerotiorum*, *Verticivium albotarum*	20–100 °C; pH 0–4, pH 9–14	Not tested	[34]

a: Antifungal activity is represented by the half maximal inhibitory concentration.
